# Alpha-synuclein from patient Lewy bodies exhibits distinct pathological activity that can be propagated in vitro

**DOI:** 10.1186/s40478-021-01288-2

**Published:** 2021-11-24

**Authors:** Nicholas P. Marotta, Jahan Ara, Norihito Uemura, Marshall G. Lougee, Emily S. Meymand, Bin Zhang, E. James Petersson, John Q. Trojanowski, Virginia M.-Y. Lee

**Affiliations:** 1 Department of Pathology and Laboratory Medicine, Institute on Aging and Center for Neurodegenerative Disease Research, University of Pennsylvania School of Medicine, Philadelphia, PA 19104 USA; 2 Department of Chemistry, University of Pennsylvania School of Arts and Sciences, Philadelphia, PA 19104 USA

## Abstract

Lewy bodies (LBs) are complex, intracellular inclusions that are common pathological features of many neurodegenerative diseases. They consist largely of aggregated forms of the protein alpha-Synuclein (α-Syn), which misfolds to give rise to beta-sheet rich amyloid fibrils. The aggregation of monomers into fibrils occurs readily in vitro and pre-formed fibrils (PFFs) generated from recombinant α-Syn monomers are the basis of many models of LB diseases. These α-Syn PFFs recapitulate many pathological phenotypes in both cultured cells and animal models including the formation of α-Syn rich, insoluble aggregates, neuron loss, and motor deficits. However, it is not clear how closely α-Syn PFFs recapitulate the biological behavior of LB aggregates isolated directly from patients. Direct interrogation of the cellular response to LB-derived α-Syn has thus far been limited. Here we demonstrate that α-Syn aggregates derived from LB disease patients induce pathology characterized by a prevalence of large somatic inclusions that is distinct from the primarily neuritic pathology induced by α-Syn PFFs in our cultured neuron model. Moreover, these LB-derived aggregates can be amplified in vitro using recombinant α-Syn to generate aggregates that maintain the unique, somatic pathological phenotype of the original material. Amplified LB aggregates also showed greater uptake in cultured neurons and greater pathological burden and more rapid pathological spread in injected mouse brains, compared to α-Syn PFFs. Our work indicates that LB-derived α-Syn from diseased brains represents a distinct conformation species with unique biological activities that has not been previously observed in fully recombinant α-Syn aggregates and demonstrate a new strategy for improving upon α-Syn PFF models of synucleinopathies using amplified LBs.

**Supplementary Information:**

The online version contains supplementary material available at 10.1186/s40478-021-01288-2.

## Introduction

Lewy Bodies (LBs) are insoluble, intraneuronal protein inclusions first characterized over 100 years ago that have long been considered the pathological hallmarks of Parkinson’s Disease (PD) [1, 2] as well as prominent pathological features of other neurodegenerative diseases of the central nervous system (CNS). Patients diagnosed with Parkinson’s Disease Dementia (PDD), Dementia with Lewy Bodies (DLB), and Alzheimer’s Disease (AD) exhibit varying degrees of LB pathology [3, 4]. LBs are structurally complex, heterogeneous inclusions consisting of many different protein components as well as organelles such as mitochondria, or parts thereof. However, one of the primary components that defines LBs is the aggregated form of the protein alpha-Synuclein (α-Syn) [5]. α-Syn is highly expressed in CNS neurons and localizes to pre-synaptic termini where it plays a role in vesicle maintenance, recycling, and release [6]. Under native conditions, cytosolic α-Syn lacks persistent secondary structure, but adopts a stable alpha-helical conformation upon binding to lipid membranes [7, 8]. However, monomeric α-Syn can also undergo spontaneous aggregation to form beta-sheet-rich amyloid fibrils and it is this fibrillar form that is a key component of LBs, Lewy neurites (LNs) as well as inclusions in other synucleinopathies such as glial cytoplasmic inclusions (GCI) in Multiple System Atrophy (MSA) [5, 9]. While insoluble α-Syn can be present in non-LB aggregates, the discovery of aggregated α-Syn in patient LBs, combined with genetic evidence linking α-Syn locus duplication and triplication, as well as α-Syn point mutants, to familial PD has solidified the central pathogenic importance of α-Syn aggregation in the formation and spread of LB pathology in the brain [10].

Monomeric α-Syn readily undergoes aggregation in vitro to form amyloid fibrils that appear to resemble those isolated from LBs [11], and these pre-formed fibrils (PFFs) are the basis of many of the models used to study LB diseases. Exogenous treatment of either primary mouse neurons or human cells with α-Syn PFFs induces the aggregation of endogenous α-Syn into inclusions that bear many of the hallmarks of patient LBs and LNs such as phosphorylation of α-Syn at Serine 129 (Pα-Syn) and co-localization with ubiquitin and p62 [12, 13]. Injection of α-Syn PFFs into the striatum of wild-type (WT) mice leads to the development of α-Syn pathology that spreads to anatomically interconnected brain regions. The progression of pathology is also accompanied by cell death and development of motor deficiencies [14]. PFFs are a powerful tool for understanding the formation and development of α-Syn aggregates, however, we sought to investigate how closely PFFs replicate the behavior of LB-derived α-Syn aggregates as, thus far, direct observation of biological activity of LB-derived aggregates is limited. α-Syn containing LBs can be isolated from pathological inclusions from patient brains using detergent-dependent, sequential extraction, but contaminant proteins make biochemical characterization of aggregates challenging. Furthermore, LBs are cytotoxic at high doses [15]. Injections of mice or primates with GCI or LB extracts have shown inclusion formation but they were often limited by low α-Syn concentrations in extracts [16, 17].

Here we were able to demonstrate that LB α-Syn extracted from the brains of both PDD and AD patients with extensive LB pathology exhibit a pathological phenotype in primary mouse neurons that is unique from that induced by recombinant α-Syn PFFs. Compared to PFFs, patient-derived LB α-Syn induced pathology that was different in both the abundance and cellular distribution, with a striking preference for large somatic inclusions. This phenotype was consistent from LBs isolated across cases for both AD and PDD. To our knowledge, this work represents the first demonstration of robust α-Syn pathology induced in neurons by patient-derived LB α-Syn and the first demonstration of a unique, LB-specific pathological phenotype that is different from recombinant PFF induced pathology.

Due to the complications of working with brain extracts, we sought to enrich the LB derived aggregates by incubation with recombinant α-Syn monomer in a seed-templated amplification. We found that these passaged LB fibrils consistently replicated the pathological phenotype of the original α-Syn LB seeds in cultured neurons. Amplification of LB α-Syn fibrils that we refer to here as LB-P1 allowed us to both inject a higher dose of LB-P1 material into WT mouse brains than could be achieved with neat LB extracts, as well as to incorporate a fluorescent BODIPY tag to facilitate observations of fibril uptake in live neurons. In both models, LB-P1 from all cases were consistently different from α-Syn PFFs. Taken together, these results demonstrate that α-Syn aggregates derived from patient LBs induce a cellular response that is distinct from recombinant α-Syn PFFs, and that these differences persist even after amplifying LB seeds with recombinant α-Syn monomer.

## Methods

### Preparation of sarkosyl-insoluble LB α-Syn from disease and control brains

**Fig. 1:**
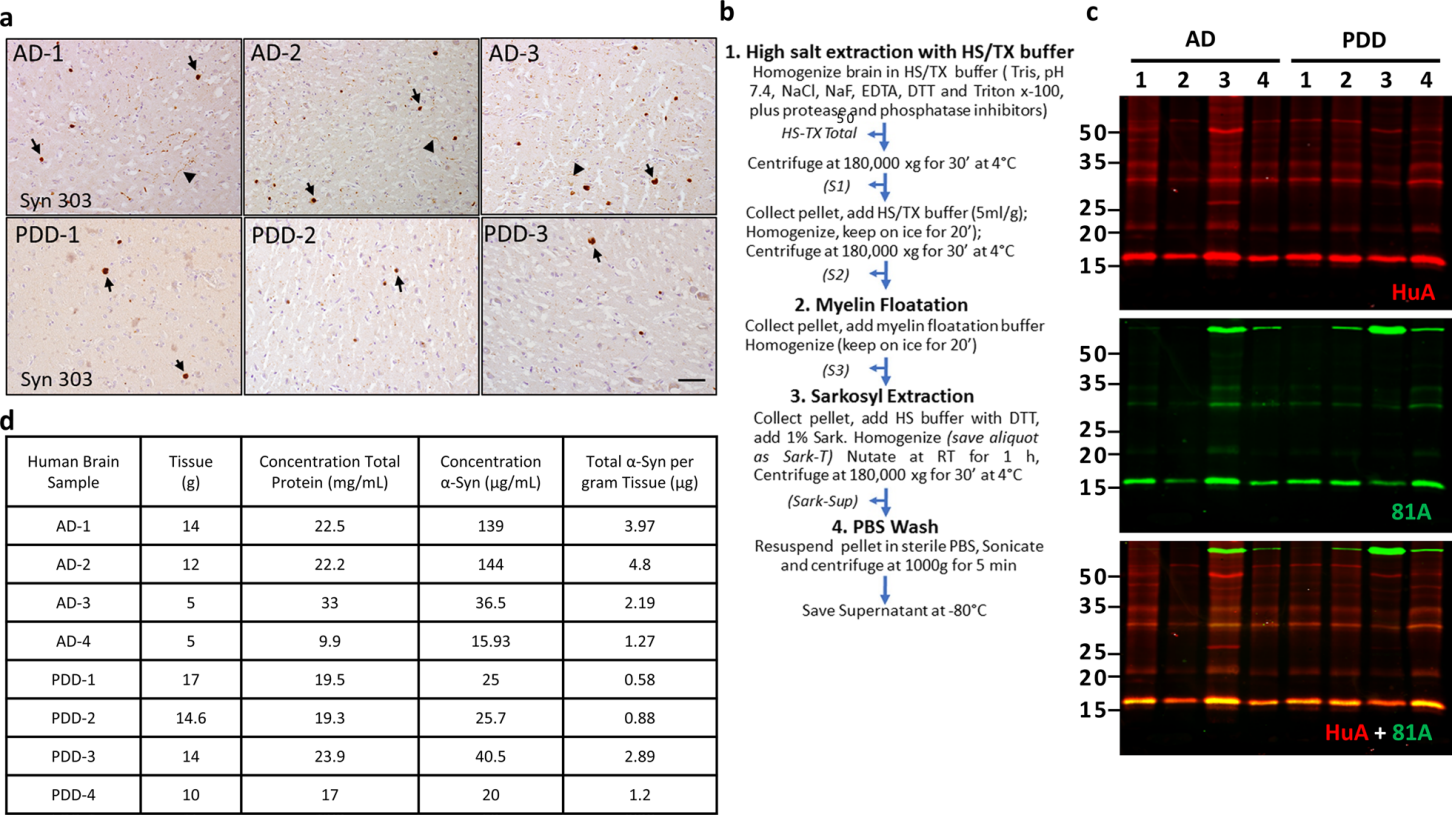
Purification and characterization of Frontal Cortex Lewy Bodies (LB) from AD and PDD brains. **a** Immunohistochemistry images of α-Syn positive LBs (arrows) and LNs (arrowheads) in frontal cortex of postmortem brain samples of 3 of the AD and 3 of the PDD patients used for this study before sequential extraction and purification. Scale bar 50 µm. **b** Schematic representation of the sequential extraction procedure used to purify pathological α-Syn from frontal cortex of postmortem human brain tissue. **c** Western blot analysis of purified Sarkosyl-insoluble α-Syn in AD and PDD brains probed with total α-Syn (HuA) and Pα-Syn (81A) antibodies. **d** ELISA and BCA analysis of purified pathological α-Syn in AD and PDD brains

All procedures were performed in accordance with local institutional review board guidelines. Written informed consent for autopsy and analysis of tissue sample data was obtained either from patients themselves or their next of kin. Frozen postmortem human frontal cortex brain tissues were selected for sequential extraction of LB and LN α-Syn based on a high burden of α-Syn pathology determined by immunohistochemical staining using previously established methods [18]. In brief, 5–10 g of frontal cortical gray matter were homogenized in five volumes (W/V) of high-salt (HS) buffer (50 mM Tris–HCl pH 7.4, 750 mM NaCl, 10 mM NaF, 5 mM EDTA) with protease and protein phosphatase inhibitors, incubated on ice for 20 min and centrifuged at 100,000 ×g for 30 min (Fig. 1b). The pellets were then re-extracted with HS buffer, followed by sequential extractions with five volumes of 1% Triton X-100-containing HS buffer and 1% Triton X-100-containing HS buffer with 30% sucrose. The pellets were then re-suspended and homogenized in 1% sarkosyl-containing HS buffer, rotated at room temperature for 2 h or at 4 °C overnight and centrifuged at 100,000 ×g for 30 min. The resulting sarkosyl-insoluble pellets were washed once with Dulbecco’s PBS (DPBS) and re-suspended in DPBS by sonication (QSonica Microson XL-2000; 50 pulses; setting 2; 0.5 s per pulse). This suspension was termed the ‘sarkosyl-insoluble fraction’, which contained pathological α-Syn and was used for the cellular and in vitro assays described here. These final sarkosyl-insoluble fractions are referred to in the text as “brain extracts” or “extracts” interchangeably. The amount of α-Syn, tau, Aβ40 and Aβ42 in the sarkosyl-insoluble fractions was determined by sandwich ELISAs (see ‘Sandwich ELISA’) and the protein concentrations were examined by bicinchoninic acid (BCA) assay.

### Sandwich ELISA

The concentrations of tau, Aβ 1–40 and Aβ 1–42 in the sarkosyl-insoluble fraction of human brain extractions were measured using sandwich ELISA as previously described [19], with the following combinations of capture and reporting antibodies: Tau5/BT2^+^HT7 for tau [20], Ban50/BA27 for Aβ 1–40, Ban50/BC05 for Aβ 1–42 [21, 22]. To measure the concentration of LB α-Syn, 384-well Nunc Maxisorp clear plates were coated with 100 ng (30 μl per well) Syn9027, a monoclonal antibody (Mab) to α-Syn, in sodium carbonate buffer, pH 9.6 and incubated overnight at 4 °C. The plates were washed 4 × with PBS containing 1% Tween 20 (PBS-T), and blocked using Block Ace solution (90 μl per well) (AbD Serotec) overnight at 4 °C. Brain lysates were sonicated (10 min., 30 s on, 30 s off, 10 °C, high setting, Diagenode Biorupter™), serially diluted in PBS and added to each well. The plates were incubated overnight at 4 °C. The recombinant α-Syn monomer and α-Syn PFFs were used as standards. The plates were then washed with PBS-T and a rabbit anti-α-Syn Mab, MJFR1 (1:1000, 30 μl per well) or a polyclonal pan-α Syn-specific antibody (HuA, 1:2000) was added to each well and incubated at 4 °C overnight. After washing, goat-anti-rabbit-IgG conjugated to horse radish peroxidase (Cell Signaling Technology, 1:10,000, 30 μl per well) was added to the plates followed by incubation for 1 h at 37 °C. Following another wash, the plates were developed for 10–15 min using 1-Step Ultra TMB-ELISA substrate solution (Thermo Fisher Scientific, 30 μl per well), the reaction was quenched using 10% phosphoric acid (30 μl per well) and plates were read at 450 nm on a Molecular Devices Spectramax M5 plate reader.

### Recombinant α-Syn purification and in vitro PFF fibrillization

Purification of recombinant human α-Syn and generation of α-Syn PFFs was conducted as described elsewhere [12, 13]. Full-length human α-Syn (1–140) proteins were expressed in BL21 (DE3) RIL competent *E. coli* and purified as previously described [23]. Fibrillization into α-Syn PFF was conducted by diluting recombinant α-syn to 5 mg/ml in sterile DPBS (Cellgro, Mediatech; pH 7.4) followed by incubating this recombinant α-Syn at 37 °C with constant agitation at 1000 rpm for 7 days. Successful α-Syn fibrillization was verified by sedimentation assay.

### Western blotting

For western blotting, 30–50 ng of sarkosyl-insoluble LB α-Syn fractions from each patient’s brain was isolated by sodium dodecyl sulfate polyacrylamide gel electrophoresis (SDS-PAGE) using NuPAGE Novex 12% Bis–Tris gels and transferred to 0.22 μm nitrocellulose membranes. The nitrocellulose membranes were then blocked in Odyssey blocking buffer (Li-Cor Biosciences, Cat. 927–40,000) diluted in Tris-buffered saline (TBS) at room temperature for 1 h. After blocking, the membranes were probed with HuA (1:2000), and 81A, a pS129 α-Syn-specific antibody (1:1000). Primary antibodies were detected using IRDye 800 (Li-Cor 925-32210) and IRDye 680 (Li-Cor 925-68071) labeled secondary antibodies, scanned on a Li-Cor Odyssey Imaging System and analyzed using Image Studio software (Li-Cor Biosciences).

### In vitro amplification of α-Syn from brain extracts

LB α-Syn from brain extracts of patients pathologically confirmed with either AD or PDD were sonicated on high for 20 cycles of 30 s on, followed by 30 s off (Diagenode Biorupter UCD-300 bath sonicator). The amplification reaction was set up with 5% LB α-Syn (calculated based on sandwich ELISA) and 95% α-Syn monomer and incubated in DPBS (pH 7.4, without Mg^2+^ and Ca^2+^) at 37 °C with constant agitation at 1000 rpm. The samples were incubated for 4–14 days in a 50–100 μl volume and the resulting samples were termed passage 1 (P1). Amplification progress was determined by removing a 2 μl aliquot and diluting it tenfold in DPBS to a final α-Syn concentration of 10 ng/μl. Samples were centrifuged at 100,000 ×g for 30 min at 24 °C in a TLA-55 rotor (Beckman Coulter). The supernatants were carefully removed, and the pellets were suspended in 20 µl of DPBS. Equal volumes of supernatant and pellet fractions were analyzed on SDS-PAGE gels and Western blot as above. After blocking, the membranes were probed with rabbit anti-α-syn antibody (HuA) and IRDye- labeled secondary antibodies and scanned using ODY-2816 Imager (Li-Cor Biosciences).

### Primary cell cultures and α-Syn treatment

Primary mouse neurons were prepared from the hippocampus of embryonic day E16–E18 CD1 mouse embryos as previously described [13, 24]. All procedures were performed according to the National Institutes of Health Guide for the Care and Use of Experimental Animals and were approved by the University of Pennsylvania Institutional Animal Care and Use Committee. Dissociated hippocampal neurons were plated at 100,000 cells/well (24-well plate) or 17,500 cells/well (96-well plate) in neuron media (Neurobasal medium, Thermo Fisher 21,103,049) supplemented with B27 (Thermo Fisher 17,504,044), 2 mM GlutaMax (ThermoFisher 35,050,061), and 100 U/ml penicillin/streptomycin (ThermoFisher 15,140,122).

PFFs, patient derived LB α-Syn fractions, or amplified LB-P1 were diluted in DPBS and sonicated on high for 20 cycles of 30 s on, followed by 30 s off (Diagenode Biorupter UCD-300 bath sonicator). Neurons were then treated with noted dose of sonicated PFFs, α-Syn sarkosyl-insoluble fractions, LB-P1, or PBS at 7 days in vitro (DIV). For chloroquine treated neurons, cells were treated with a 25uM chloroquine pulse 6 h post-treatment for 30 min, followed by two washes with conditioned neuronal media. Neurons were fixed and immunostained at 14 days post-treatment (21 DIV) unless otherwise indicated.

### Immunocytochemistry and quantification of neuron pathology

Primary neuron cultures were washed with PBS once and fixed at DIV 21 with ice-cold methanol for 20 min in a −20 °C freezer (24 well plates), or fixed with 4% paraformaldehyde, 4% sucrose, and 1% Triton-X100 in PBS (96 well plates). After PBS washes, cells were blocked with 3% BSA, 5% FBS in DPBS for 1 h at room temperature, then incubated with primary antibodies (Additional file 8: Table 1) at 4 °C overnight. Cells were washed 5 times with PBS and incubated with secondary antibodies for 2 h at room temperature. After washing with PBS 5 times, coverslips from 24 well plates were mounted with Fluoromount G with DAPI (eBioscience) and scanned on a Perkin Elmer Lamina scanner. The total amount of 81A and MAP2 signals for neuronal cultures and the total number of DAPI-positive nuclei were quantified using Indica Labs HALO software.

**Table 1:** Demographics of patient brains with α-Synucleinopathies

Case ID	Gender	Disease duration (years)	Age at death	PMI (hrs)	Neuropathological diagnosis-1	Neuropathological diagnosis-2
AD-1	M	6	74	19	AD	LBD, DLB
AD-2	F	11	70	18	AD	LBD, DLB
AD-3	M	8	72	8	AD	LBD, DLB
AD-4	F	12	72	4	AD	PSP, LBD, DLB
PDD-1	F	29	73	30	PDD	AD
PDD-2	M	21	75	6.5	PDD	–
PDD-3	M	18	59	10	PDD	–
PDD-4	M	5	83	9	LBD	AD
PDD-5	F	18	86	20	PDD	AD

All human synucleinopathy cases used in the study, gender, disease duration (in years), age of death, and post-mortem interval (PMI) (in hours)

For cells cultured in 96 well plates, the cells were washed and incubated in DAPI solution (ThermoFisher Cat#D21490, 1:10,000 in DPBS) after staining with secondary antibodies and the plates were sealed with adhesive covers. The 96 well plates were scanned with an In Cell Analyzer 2200 (GE Healthcare) with a 10 × or 40 × objective and analyzed using the accompanying software (In Cell Toolbox Analyzer). Quantitation of total 81A signal and the amount of somatic 81A signal was calculated using Cell Profiler ver. 3.1.9 (The Broad Institute). Nuclei were identified by thresholding the DAPI signal (three class Otsu) and discarding objects outside a 12–40 pixel diameter range. Identified nuclei were used to define MAP2 positive secondary objects referred to as cell bodies. MAP2 signal threshold was determined (three class Otsu, 0.75 correction factor) and cell bodies were defined (Distance-B method) as extending up to 10 pixels from the seeding nucleus within the MAP-2 area [25]. The threshold for 81A signal was determined (robust background; lower outlier fraction 0.1, upper outlier fraction 0.01) and objects greater than 60 pixels in diameter were discarded. 81A positive objects larger than 200 square pixels in area with an eccentricity less than 0.95 (0 for perfectly round, 1 for straight line) that overlap with a DAPI positive, MAP2 positive cell body were classified as somatic inclusions. All other 81A positive objects were considered to be neuritic. Total 81A object area was normalized to the DAPI count for each wells. The fraction of somatic inclusions was calculated as the 81A signal intensity (density times area) of somatic objects divided by the total 81A signal intensity. For each well, values were summed across 12 separate, tiled images covering 76% of the well. Data are reported as the average of at least 3 replicate wells for each treatment sample. Standard deviations of replicate wells were calculated and statistical significance was determined in Graphpad Prism (GraphPad Software) by unpaired t-test (two-tailed) with Walsh’s correction.

### Proteinase K digestion of sarkosyl-insoluble LB- α-Syn and amplified samples from AD and PDD brains

Sarkosyl-insoluble LB α-syn fractions from the frontal cortex of AD and PDD brains, including amplified P1 and P2 samples, were prepared as described above. For proteinase K (PK) digestion, equal amounts (300 ng) of α-syn from each sample were sonicated 20 cycles of 30 s on 30 s off as described above and mixed with 0.25 μg of PK in DPBS to a final volume of 30 μL and incubated at 37 °C for 10 min. The reaction was stopped with 1 mM PMSF. Reaction samples were boiled with SDS-sample buffer for 10 min and resolved on NuPAGE Novex 12% Bis–Tris gels (Invitrogen). The samples were transferred from 12% Bis–Tris gel to 0.22 μm nitrocellulose membranes, and the resulting α-Syn fragments were analyzed by immunoblotting using anti α-Syn antibody (HuA). Densitometry of bands was performed in ImageJ (NIH).

### Preparation of fluorophore-labeled LB α-synuclein

BODIPY-labelled α-Syn was prepared as described previously [26, 27]. Briefly, E114C mutant α-Syn was expressed in BL21 (DE3) *E.coli* and purified using Ni–NTA resin (Gold Biotech), dialyzed, and alkylated with BODIPY-Fl maleimide (Lumiprobe). The labelled protein was purified over a HiTrap Q HP column on an AKTA FPLC (GE Life Sciences) and characterized by MALDI-TOF mass spectrometry. To generate our 25% fluorescently labelled aggregates, 70 ng/µL WT monomer plus 25 ng/µL of BODIPY-E114C monomer were incubated with either 5 ng/µL PFF or 5 ng/µL of patient derived LB α-Syn and amplified as described above.

#### Uptake of fluorescently labelled α-Syn in live neurons

Primary mouse hippocampal neurons were cultured in 96 well plates as described above. At 7 DIV, neurons on five replicate 96 well plates were treated with 100 ng of 25% BODIPY-labelled α-Syn seeded with either PFF (BODIPY-PFF) or patient LB (BODIPY-LB-P1) from extracts AD1-3 and PDD1-3. Four replicate wells were treated for each sample, for each replicate plate. Replicate plates were imaged at 2, 6, 12, 24, or 36 h post-treatment on an In Cell Analyzer 2200 (GE Healthcare) with a 40 × objective. Cells were treated with media containing NucBlu dye (Life Technologies) 30 min prior to imaging to label nuclei. Media was removed and replaced with 10 mM HEPES in HBSS (Invitrogen) containing 500 mM Trypan Blue (Sigma Aldrich) 5 min before imaging to quench all extracellular BODIPY fluorescence. For each well, values were summed for 9 images evenly distributed across the well. Data is reported as the average of 4 replicate wells for each treatment sample for each time point. Standard deviations of replicate wells were calculated, and statistical significance compared to BODIPY-PFF was determined in Graphpad Prism (GraphPad Software) for each BODIPY-LB-P1 sample at each time point by multiple t-test.

#### Animals and ethics statement

C57BL/6 C3H (B6C3) female mice at 2 months of age purchased from Charles River were used for the experiments. All animal procedures were approved by the University of Pennsylvania Institutional Animal Care and Use Committee and conformed to the National Institute of Health Guide for Care and Use of Laboratory Animals.

#### Stereotaxic inoculation of WT mice with amplified LB-P1 or PFF α-Syn

Stereotaxic surgery was performed as described previously with minor modifications [14]. Mice anesthetized with ketamine–xylazine–acepromazine underwent stereotaxic injections using a 33 gauge syringe. Mice received a unilateral injection of 2.5 μl of amplified LBs (200 ng/μl, 500 ng total dose) or human α-Syn PFFs in PBS (2 μg/μl, 5 µg total dose) into the dorsal striatum (coordinates: 0.2 mm relative to bregma; 2.0 mm from midline; – 3.2 mm beneath the skull surface). These mice were sacrificed at 6 months post-injection (3 animals per treatment). Following perfusion with 4% (w/v) paraformaldehyde (PFA), the brains were collected and immersed in 4% PFA at 4 °C overnight. Tissues were then embedded in paraffin for sectioning.

#### Immunohistochemical analysis of injected mouse brains

Six-μm thick paraffin sections were prepared for immunohistochemical analyses. To assess the distribution of α-Syn pathology, every 20th paraffin section throughout the brains was incubated with anti-Pα-Syn antibody EP1536Y (Abcam, #ab51253, 1:20,000) at 4 °C for 2 days and then processed for visualization. An anti-rabbit biotinylated secondary antibody (Vector laboratories) was used for generating diaminobenzidine reaction product. For each treatment, the total number of neurons with cell body inclusions was determined and averaged across 3 animals. Standard deviations of replicate animals were calculated for each time point and statistical significance was determined in Graphpad Prism (GraphPad Software) for LB-P1 injected versus PFF injected by unpaired t-test (two tailed). Semi-quantitative analyses were performed for Pα-Syn positive pathology on the six coronal sections (2.80, 1.78, 0.26, −1.58, −2.92, and −4.04 mm relative to bregma), and color coded onto heat maps. The extent of Pα-Syn-positive pathology was graded as 0–3 (0, no pathology; 0.5, mild; 1, moderate; 2, severe; 3, very severe) based on the criteria reported previously [28]. Sections were examined with a BX43 microscope (Olympus).

## Results

### Characterization of frontal cortex Lewy bodies (LBs) from AD and PDD brains

Cases of AD and PDD (Table 1) used for extraction of LB α-Syn were selected based on the abundance of α-Syn LB pathology. The frontal cortex was selected from each case for extraction so that larger amounts of brain tissue were available to yield sufficient pathological α-Syn in the final lysate to perform multiple experiments. The overall burden of LB α-Syn in the frontal cortex of each case was first evaluated by immunohistochemistry using an anti- α-Syn antibody (Syn303) that intensely stains intraneuronal LBs and LNs (Fig. 1a). The presence of LB and LNs in these fractions was further confirmed by the immunodetection of Pα-Syn as well as ubiquitin, and p62 (Additional file 1: Fig. S1), all of which are known components of LBs.

The cortical samples underwent sequential extraction to enrich for insoluble, pathological LB α-Syn (Fig. 1b). Western blot analysis with a polyclonal pan-α-Syn antibody (HuA), as well as a Pα-Syn-specific antibody (81A) showed the successful extraction of α-Syn aggregates from AD and PDD brain lysates. We observed a very strong total α-Syn (HuA) and Pα-Syn (81A) signal in sequentially extracted AD and PDD brain lysates at molecular weights corresponding to monomeric α-Syn (15KD) (Fig. 1c). The concentration of insoluble α-Syn in AD and PDD brain lysates was determined by sandwich ELISA using the monoclonal anti-α-Syn antibody (9027) for capture and rabbit polyclonal HuA or rabbit monoclonal MJFR1 for detection. Concentrations of recombinant human α-Syn PFF and human α-Syn monomer ranging between 10 and 100 ng/ml were used for the standard curve. The results of the ELISA are shown in Fig. 1d. Overall, the amount of total α-Syn detected in the insoluble fractions was between 0.6 and 4.8 µg per gram of brain tissue.

### LB α-Syn extracts from human AD or PDD brains induce robust α-Syn pathology in mouse primary hippocampal neurons

**Fig. 2:**
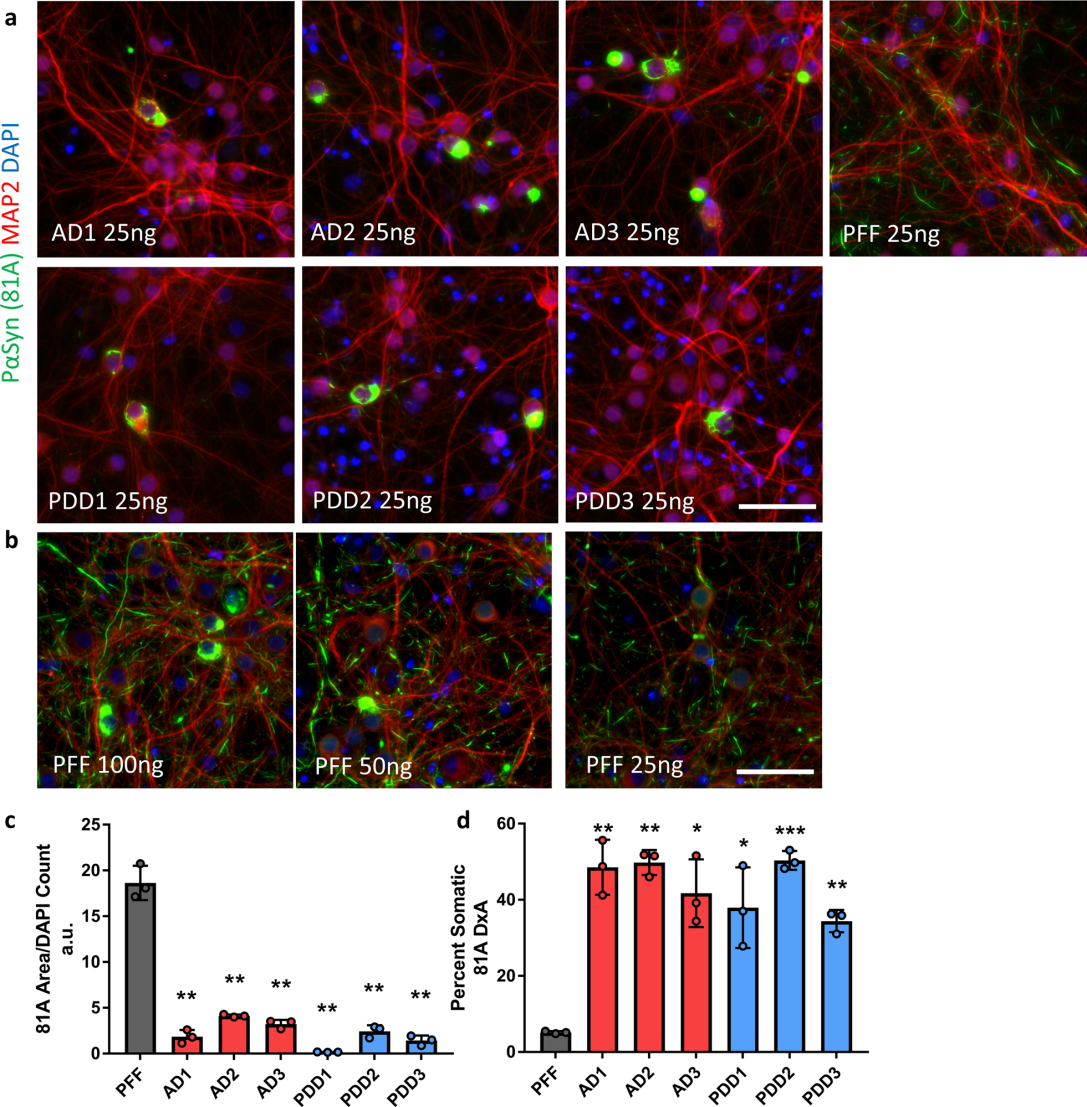
Patient-derived, insoluble α-Syn species are active in cultured mouse hippocampal neurons and induce distinct α-Syn pathology. **a** Primary hippocampal neurons were treated in 96 well plates at DIV 7 for 14 days with 25 ng of PFF, AD or PDD α-Syn. AD and PDD α-Syn induced somatic aggregates in neuronal cell bodies and some thread like neuritic aggregates whereas human α-synuclein PFF’s induced predominantly neuritic aggregates. DAPI and MAP2 were used to show nuclei and somatodendritic area. Scale bars 50 µm. **b** Neurons were treated in 96 well plates at DIV 7 for 14 days with increasing amounts (25, 50, 100 ng) of human PFF. The number of somatic aggregates increased with higher concentrations of human PFFs, but neuritic pathology also increased. Scale bar 50 µm. **c** Quantification of total 81A pathological area in neurons treated in 96 well plates with 25 ng per well AD, PDD, or PFF α-Syn. **d** Percent of total 81A pathology in somatic inclusions from neurons treated as in **c**. Totals are the average of 3 replicate wells. Error bars represent the standard deviation. Significance compared to PFF treated neurons was determined by student t-test (*p < 0.05, **p < 0.01, ***p < 0.001)

To determine whether the AD and PDD derived LB α-Syn induces α-Syn pathology, we treated primary hippocampal neurons from WT mice with enriched LB α-Syn extracts from AD and PDD brains or human α-Syn PFFs and probed for the presence of insoluble Pα-Syn pathology. Primary hippocampal neurons cultured for 7 days in vitro (DIV 7) were treated for 14 days with equal amounts of PFFs and AD or PDD LB α-Syn (25 ng per well), then fixed and extracted with 4% PFA and 1% Triton-X100 followed by immunostaining with 81A and imaged. At this dose, immunocytochemistry (ICC) showed the pathology induced by PFFs consisted predominantly of short, neuritic aggregates and small somatic puncta distributed evenly throughout the culture (Fig. 2a, b, Additional file 2: Fig. S2). However, the pathology induced by LB α-Syn from both AD and PDD patients exhibited distinct morphological features and distribution patterns compared to PFF-induced pathology. Importantly, LB pathology was comprised mostly of large inclusions in the perinuclear part of the soma and sometimes appeared as rods in MAP2 positive neurites that covered several dendritic branches. LB pathology was also more discrete than PFF pathology, localized to relatively few neurons each exhibiting robust pathology while the majority of surrounding neurons were spared. Finally, LB α-Syn obtained from all three AD and three PDD cases induce similar LB pathology in primary neurons (Fig. 2a, Additional file 2: Fig S2).

Next, mouse hippocampal neurons were treated with increasing concentrations of PFFs (20–1000 ng) (Fig. 2b) or with AD and PDD LB α-Syn (5–30 ng) (Additional file 3: Fig. S3). PFF treated neurons exhibited increased somatic inclusions at higher concentrations, but with an increase in neuritic pathology as well. Furthermore, primary neurons treated with 15–25 ng/ml of AD or PDD α-Syn developed many more somatic inclusions than those treated with the same amount of PFFs. We did not test higher doses of AD or PDD LB α-Syn both to conserve material and because patient derived LB extracts at high concentrations were toxic to neurons. Immunostaining of MAP2 in 14-days post treatment neurons showed reduction in the density of MAP2, whereas intact neuronal processes were observed in PFF treated neurons. This reduction of MAP2 immunostaining was likely due to cell stress caused by high levels of insoluble contaminant proteins in the brain extracts.

To better quantify the differences between LB and PFF induced α-Syn pathology, we treated hippocampal neurons cultured in 96-well plate format and analyzed Pα-Syn pathology by high content analysis. Neurons were treated at DIV 7 with 25 ng per well of AD or PDD extracts or PFF and fixed for ICC on DIV 14. Total Pα-Syn area was normalized to DAPI count and Pα-Syn-positive objects were filtered by size, shape, and co-localization with DAPI and MAP2 to determine the percent of total 81A signal in somatic inclusions. The total Pα-Syn pathology for all LB samples was less abundant compared to PFF (Fig. 2c), but the proportion of pathology present as somatic inclusions was significantly higher than PFF for all LB samples tested (Fig. 2d). While total pathology varied amongst LB cases, the amount of somatic pathology induced by LB treatment was not dependent on the total pathological activity of a given case or the disease of origin, with all cases inducing a similarly high percentage of somatic inclusions. This reinforces the specificity of this pathological morphology for differentiating LB α-Syn from other aggregates.

To further characterize the induction and maturation of LB α-Syn induced pathology, primary neurons were treated in 24-well plates with equal amounts of PFFs or AD and PDD LB α-Syn at DIV 7 for 3, 7 and 14 days. AD and PDD LB α-Syn induced pathology in a time dependent manner (Additional file 4: Fig. S4). We detected Pα-Syn accumulation as early as 3 days post treatment with AD and PDD LB α-Syn. At day 3, pathology was very sparse, but somatic inclusions were already observable. At day 7, neurons started to exhibit increased Pα-Syn 81A somatic inclusions. At day 14, the number of somatic inclusions continued to increase and the filamentous Pα-Syn pathology in MAP2 + neurites were more prominent. Very little Pα-Syn staining was observed at day 3 in PFF treated neurons. Pathology was observed only in neurites and as puncta in neurons treated with PFF at days 3, 7, and 14.

### Seed-templated amplification of AD, PDD aggregates in vitro recapitulates pathological phenotype

**Fig. 3:**
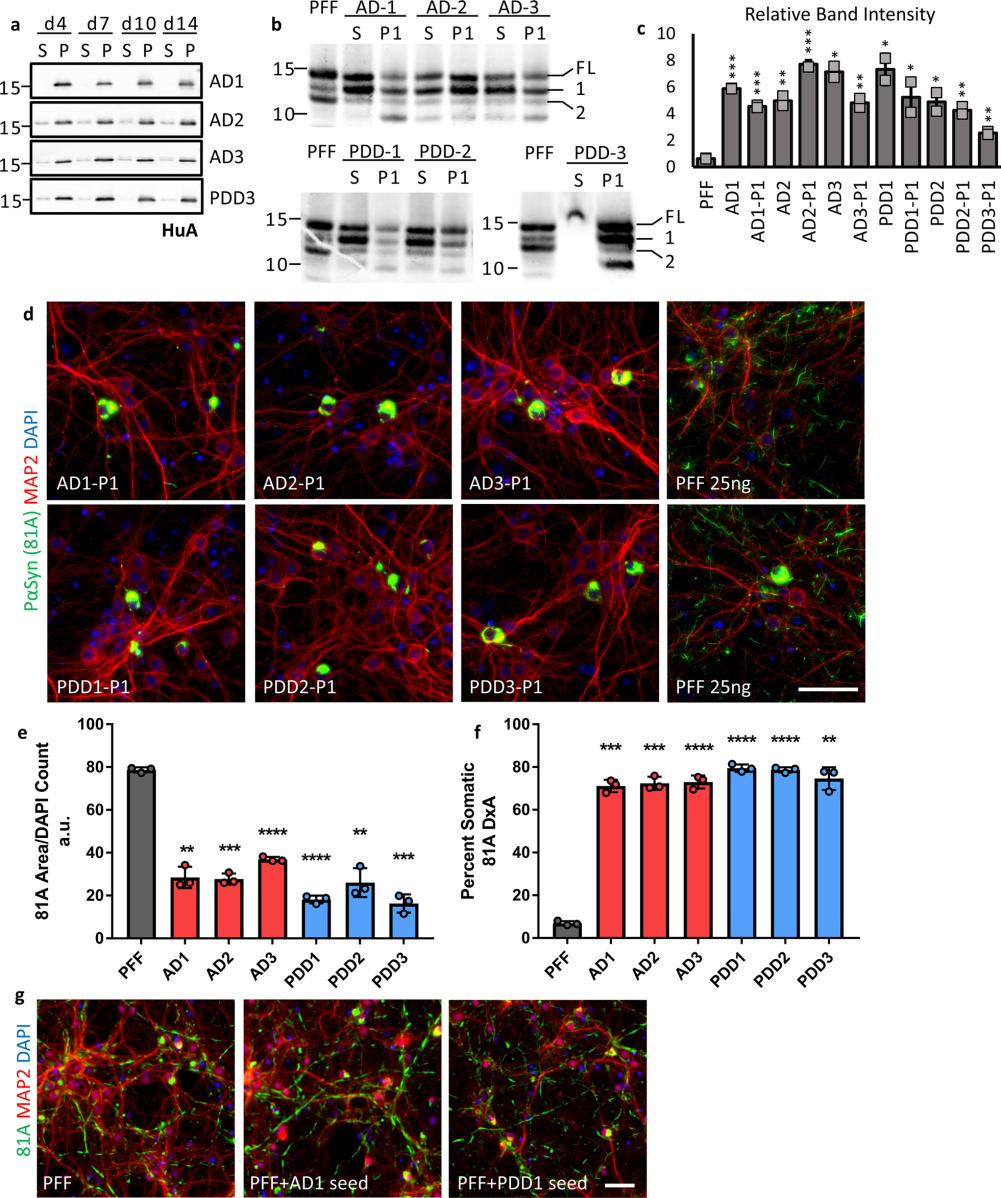
In vitro amplification of patient LB α-Syn preserves characteristic pathological phenotype of the original material. **a** Aggregation of recombinant monomer seeded with 5% patient LB α-Syn was monitored by sedimentation. Aliquots were taken from aggregation reactions at 4, 7, 10, and 14 days and centrifuged at 100 k x g for 30 min at room temperature. Supernatant (S) and pellet (P) fractions were analyzed by Western blot. Membranes were probed with HuA. **b** 300 ng of LB seeds (S) or LB-P1 (P1) was partially digested with 0.25 ug of Proteinase K (PK) for 10 min at 37 °C. The resulting PK resistant bands were analyzed by Western blot. Membranes were probed with HuA. Major bands labelled full-length (FL), first fragment (1), and second fragment (2). The level of contaminant protein in PDD3 inhibited digestion of the seed sample. **c** Band intensity for fragments 1 and 2 were quantified and relative intensity of fragment 1 to fragment 2 was plotted for each sample to highlight the banding pattern. Values are the average of two independent digestions. **d** Mouse hippocampal neurons were treated in 96-well plates with 25 ng of α-Syn PFF or LB-P1 at DIV 7 for 14 days. 81A positive pathology induced by LB-P1 is consistent with LB seeds pathology. Scale bar 50 µm. **e** Quantitation of total 81A area for neurons treated as in **d**. **f** Percent of total 81A pathology in somatic inclusions from neurons treated as in **d**. Totals are the average of 3 replicate wells. **g** Mouse hippocampal neurons treated in 96 well plates with 25 ng of PFF or 95 ng PFF pre-incubated with 5 ng LB seeds to demonstrate that addition of LB seeds to PFF does not induce primarily cell body inclusions. PFF + seed samples were incubated the same as for P1 reactions. Scale bar 50 µm. All error bars represent the standard deviation. Significance compared to PFF was determined by student’s t-test (*p < 0.05, **p < 0.01, ***p < 0.001, ****p < 0.0001)

Despite showing pathological activity, the contaminant proteins in our brain extracts induced significant toxicity in cultured neurons and limited their usefulness in further experiments. To overcome this challenge and to improve the consistency of our neuronal model, we sought to amplify the α-Syn aggregates present in our LB extracted samples using a seed-dependent propagation strategy. α-Syn monomers form fibrils in a two-step, nucleation-dependent manner, with unstructured monomers first forming a beta-sheet rich nucleus that is then elongated by the addition of monomers to the ends of the nascent fibril [11]. The nucleation process is slow and the kinetics of fibril formation are greatly accelerated by the addition of a small amount of fibril fragments that act as seeds for the initiation and growth of new fibrils. Thus, we diluted our LB extracts to a final α-Syn concentration of 5 ng/µL and incubated with a 20-fold excess of recombinant α-Syn monomer at 37 °C, with constant shaking, for up to 14 days to generate new aggregates referred to hereafter as passage 1 or P1. Due to the high background associated with the contaminant proteins in our extracts, we could not monitor the progression of our amplification using the commonly employed amyloid sensitive dye Thioflavin-T. Instead, amplification progress was monitored by sedimentation at 100,000 ×g for 30 min followed by immunoblot analysis of both supernatant and pellet fractions with a polyclonal anti-α-Syn antibody (HuA) (Fig. 3a). Reactions for all cases tested showed significant pelletable α-Syn after only 4 days of incubation, reaching a maximum between 7 and 10 days of incubation. To determine if the P1 aggregates being generated have a similar core structure as the LB derived seeds, we performed a proteinase K (PK) digestion on both seeds and amplified products, generating a major band labelled fragment 1 and a minor band labelled fragment 2. Seeds and their respective P1 products all showed similar banding patterns, with fragment 1 much more intense relative to fragment 2, suggesting that they have a consistent PK-resistant fibril core (Fig. 3b). Digested PFF samples produced similar fragments 1 and 2 to LB samples, with several additional bands. The pattern of band intensities for PFF samples was different however, with fragment 2 being more intense relative to fragment 1. This differing ratio was quantified for two independent digestions and is plotted in Fig. 3c. However, there was variability between LB cases, making PK insufficient to demonstrate the fidelity of amplification in isolation.

We next sought to evaluate if the P1 species generated in our amplification reaction induced the same pathological phenotype in cultured primary neurons. Primary neurons were treated in 96-well plates with 25 ng per well of either PFF or P1 material amplified from AD or PDD extracts. We observed the same pathological phenotype that was induced by the primary LB α-Syn (Fig. 3d, Additional file 5: Fig S5) with large somatic inclusions being the predominant form of inclusions, with little neuritic pathology. In order to quantify the similarity of the LB-P1 induced pathology to the original LB seeds, primary mouse neurons were cultured in 96-well plates and triplicate wells treated with 25 ng each of LB-P1 generated from AD case 1, 2, or 3 or PDD case 1, 2, or 3. We then performed the same high content analysis used for the LB seeds samples. Again, we found that the total pathology induced by P1 samples was less than the same dose of PFF (Fig. 3e) while all LB-P1 samples showed a high percentage of total Pα-Syn contained in somatic inclusions, as defined by the same size, shape, and DAPI/MAP2 positivity filters used for the original LB samples (Fig. 3f). That this pathology was similar to the pathology induced by LB seeds alone reinforces that this pathological phenotype is specific to LB derived α-Syn. That the observed pathology is similar across all P1 samples demonstrates that the unique conformation or conformations responsible for this phenotype can be reliably propagated in a seed-dependent manner with recombinant monomers despite variability in seed concentrations of α-Syn or contaminant proteins.

We found that replicate LB-P1 products can reproducibly induced somatic pathology after 7–10 days of amplification. However, we also confirmed that the pathology induced by P1 aggregates was similar throughout the amplification time course. Treating neurons with aliquots taken at 4, 7, 10, or 14 days into P1 amplification induced somatic inclusions for AD and PDD seeds (Additional file 6: Fig. S6). Furthermore, treatment of neurons with 25 ng of PFF was compared to treatment of neurons with 95 ng of PFF plus the same amount of lysate containing 5 ng of AD1 or PDD1 seeds that was used to generate P1. While AD1-P1 and PDD1-P1 treated neurons exhibit the expected cell body pathology (Fig. 3d, Additional file 5: Fig. S5), the PFF + AD1-seeds and PFF + PDD1-seeds samples were indistinguishable from PFF treated controls (Fig. 3g). This demonstrates that our amplification reactions are successful in capturing the biological activity of the LB seeds in the P1 products and that the observed cell body pathology is due to the α-Syn aggregates present in the sample, not other components present in the LB extracts. If some other component of the seeding lysate was driving the observed LB pathology, we would expect to see similar pathology when treating with the same dose of PFF + seeds.

### Striatal injection of amplified LB-P1 aggregates into WT mice induces altered spreading of pathology

**Fig. 4:**
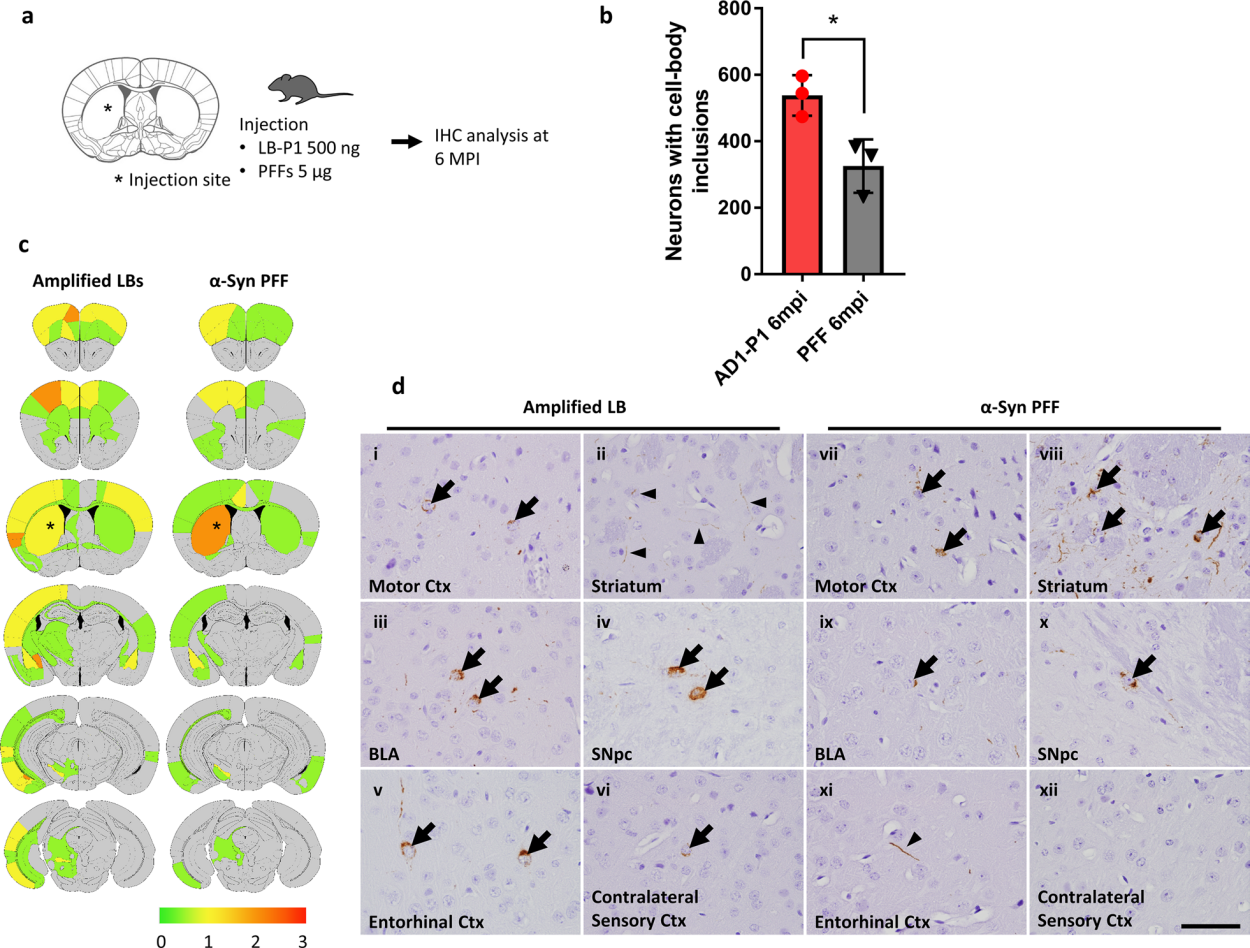
Injection of amplified LB-P1 into WT mice induces altered pathological spread compared to PFF. **a** Schematic representation of experimental design. WT mice were injected with 500 ng of LB-P1 or 5 μg of human α-Syn PFF into the dorsal striatum and analyzed by immunohistochemistry (IHC) at 6 months post-injection (mpi). **b** Quantitation of neuronal inclusion in whole brains of mice injected with LB-P1 or PFF and sacrificed 6 mpi. Totals are the average of 3 animals. Error bars represent the standard deviation. Significance compared to PFF injected animals was determined by student t-test (*p < 0.05). **c** Distribution of Pα-Syn pathology 6 mpi. Heat map colors represent the extent of pathology by semi-quantitative analysis (light yellow: mild pathology, yellow: moderate pathology, orange: severe pathology, red: very severe pathology). **d** Representative Pα-Syn immuno-histochemistry in the motor cortex (Ctx), striatum, basolateral amygdala (BLA), substantia nigra pars compacta (SNpc), entorhinal Ctx, and contralateral sensory Ctx in LBP1-injected (i–vi) and PFF–injected (vii–xii) mouse 6 mpi. Arrows indicate Pα-Syn positive, somatic inclusions. Arrowheads indicate Pα-Syn positive, neuritic inclusions. Scale bar 50 µm

Given that the cellular pathology induced by LB-P1 was highly consistent with the pathology of the LB seeds and distinct from that of α-Syn PFF, we wanted to further examine the biological activity of LB-P1 in vivo. Stereotaxic injection of α-Syn PFF into WT mice is a well-established disease model for synucleinopathy that demonstrates dose and time dependent spread of pathology from the site of injection [14]. To this end, we injected WT mice with either 500 ng of AD1-P1 or 5 μg of human α-Syn PFFs and conducted IHC analyses at 6 months post-injection (mpi) (Fig. 4a). Every 20th section of a consecutive series of paraffin embedded sections of brains were probed with the anti-Pα-Syn antibody EP1536Y (Abcam). We analyzed Pα-Syn positive pathology throughout the brains and quantified the total number of neurons with cell body inclusions (Fig. 4b). At 6 mpi, the LB-P1 injected animals exhibited a much higher number of Pα-Syn cell body inclusions than PFF injected animals. This difference is seen despite not correcting for the difference in dose between P1 and PFF (Fig. 4a). These data suggest that 500 ng of amplified LBs has a higher potency of inducing cell body pathology compared with 5 μg of PFFs, consistent with the observations in primary mouse neurons.

The spread of pathology from the site of injection was also different for LB-P1 injected animals. We performed semi-quantitative analyses for Pα-Syn positive pathology on coronal sections from 6 mpi mice and scored the extent of pathology in each region based on the criteria reported previously [28] (Fig. 4c). Heat maps comparing LB-P1 and PFF injections showed moderate to severe Pα-Syn positive pathology in several brain regions including the ipsilateral motor cortex, striatum, amygdala, substantia nigra pars compacta, entorhinal cortex, and contralateral sensory cortex. In those brain regions, with the exception of the striatum, equal or more severe pathology was observed in the amplified LB-P1-injected mice compared with the PFF–injected mice (Fig. 4d). These data also suggest that P1 induced pathology spreads more aggressively in vivo, with a comparable amount of spread observed for PFF and P1 injected animals at 6 mpi despite a tenfold difference in the injection dose.

### Uptake of Fluorescently Labelled LB-P1 in Live Mouse Hippocampal Neurons is More Robust than for labelled PFFs

**Fig. 5:**
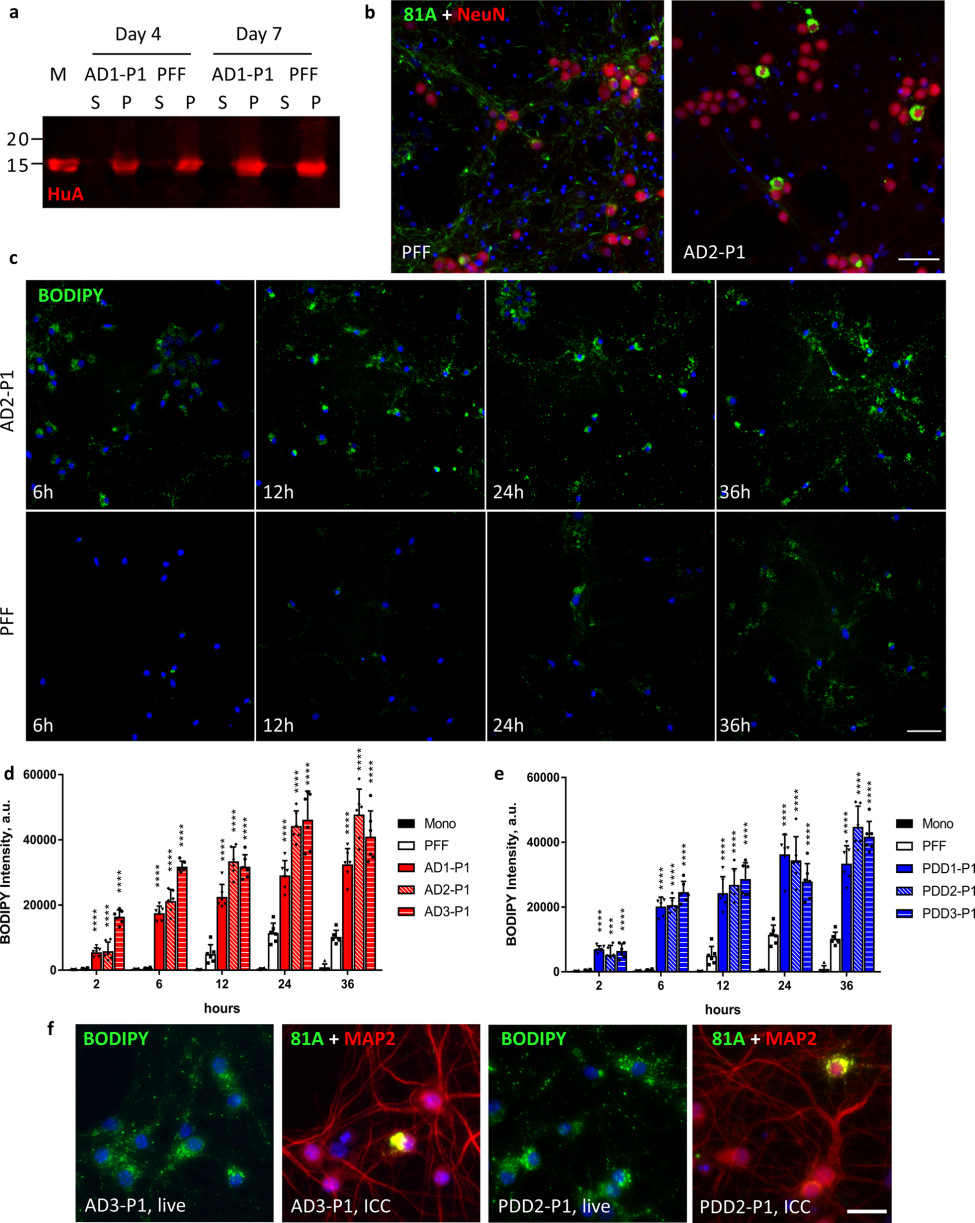
Endocytosis of BODIPY labelled LB-P1 is more rapid and more robust than labelled PFF. **a** Aggregation of recombinant monomer with 25% BODIPY-labelled E114C α-Syn and 5% LB seeds or 5% PFF was monitored by sedimentation assay. Supernatant (S) and pellet (p) fractions were analyzed by Western blot. Monomer (M) was added as a loading control. Membranes were probed with HuA. Similar kinetics were observed for LB and PFF seeded reactions. **b** Mouse hippocampal neurons were treated in 96-well plates with 50 ng of BODIPY-PFF or BODIPY-LB-P1 per well. 81A pathology observed was similar to that observed for non-labelled aggregates. Scale bar 50 µm. **c** Live mouse hippocampal neurons were treated in 96-well plates with 100 ng of BODIPY-labelled PFF or LB-P1 fibrils and imaged at 2, 6, 12, 24, or 36 h after treatment. Extracellular fluorescence was quenched with the addition of 500 mM Trypan Blue immediately before imaging. Scale bar 50 µm. BODIPY intensity of internalized, fluorescently-labelled AD-P1 **d** or PDD-P1 **e** fibrils from neurons treated as in **c**. Totals are the average of 6 replicate wells. Error bars represent the standard deviation. Significance compared to BODIPY-PFF treated neurons was determined by student t-test (*p < 0.05, **p < 0.01, ***p < 0.001, ****p < 0.0001). **f** Neurons treated in 96-well plates with 100 ng per well of BODIPY labelled AD3-P1 or PDD2-P1 and imaged live as in **b**, 24 h post-treatment. The same neurons were fixed after 14 days and ICC performed. Images show the same field of view for each treatment. Scale bar 50 µm

Given that LB-P1 induced unique pathology compared to α-Syn PFF in both in vitro in cultured neurons and in vivo in injected WT mice, we sought to further investigate differences in the cellular events upstream of pathology. Our lab and others [27, 29] have demonstrated that α-Syn fibrils are taken up predominantly through endocytosis and are trafficked to the endosomal-lysosomal pathway. However, since α-Syn fibrils are not degraded efficiently, the accumulation of fibrils in lysosomes over time leads to leakage that may allow some fibrils, or fragments thereof, to enter the cytosol and potentially template the recruitment of endogenous α-Syn to form new aggregates [27]. To test this possibility and to better understand the fate of LB-P1 fibrils upon endocytosis, we amplified P1 material from 5% LB seeds with a combination of 70% WT monomer and 25% E114C mutant α-Syn labelled with BODIPY maleimide as described previously [26, 27]. We found that the BODIPY labelled monomer was incorporated into our P1 aggregates with similar kinetics to 100% WT monomer (Fig. 5a) and that the fluorescently labelled P1 fibrils produced the same cell body inclusion as observed for 100% WT LB-P1 (Fig. 5b). With our BODIPY-labelled LB-P1 we were then able to observe the uptake of our P1 material in live mouse hippocampal neurons, as compared to 25% BODIPY labelled PFF. Neurons in 96-well plates were treated at 7 DIV with 100 ng per well of either BODIPY-PFF or BODIPY-LB-P1 and the amount of internalized BODIPY positive fibrils was observed at 2, 6, 12, 24, and 36 h post-treatment. Immediately before imaging, cells were treated with trypan blue to quench the fluorescence of the extracellular fibrils, allowing us to observe only the internalized population. Uptake of BODIPY-LB-P1 occurred earlier and was more robust at all time points compared to BODIPY-PFF (Fig. 5c). We found that all BODIPY-P1 fibrils were taken up to a much greater extent than BODIPY-PFF for both AD-P1 (Fig. 5d) and PDD-P1 (Fig. 5e). This is in agreement with our in vivo results, which suggest that LB-P1 material may spread more aggressively over time compared to α-Syn PFF. However, we did not observe any obvious difference in the subcellular localization of BODIPY positive puncta between PFF and P1 treated neurons or over the time course of the experiment (Additional file 7: Fig. S7). This difference in uptake does not, therefore, determine the difference in the distribution of pathology between PFF and P1 treated neurons. To further explore the connection between uptake and eventual pathology, we again treated neurons with BODIPY-LB-P1 and observed the uptake of BODIPY positive puncta at 24 h post-treatment. We then fixed the cells at 21 DIV (14 days post-treatment) and performed ICC (Fig. 5f). By imaging the same fields, we were able to confirm that many more cells take up the BODIPY positive fibrils than eventually develop pathology. This again suggests that uptake alone does not determine the induction of pathology by LB-P1.

### Induction of lysosomal dysfunction increases the pathology induced by LB-P1 but does not change its distribution

**Fig. 6:**
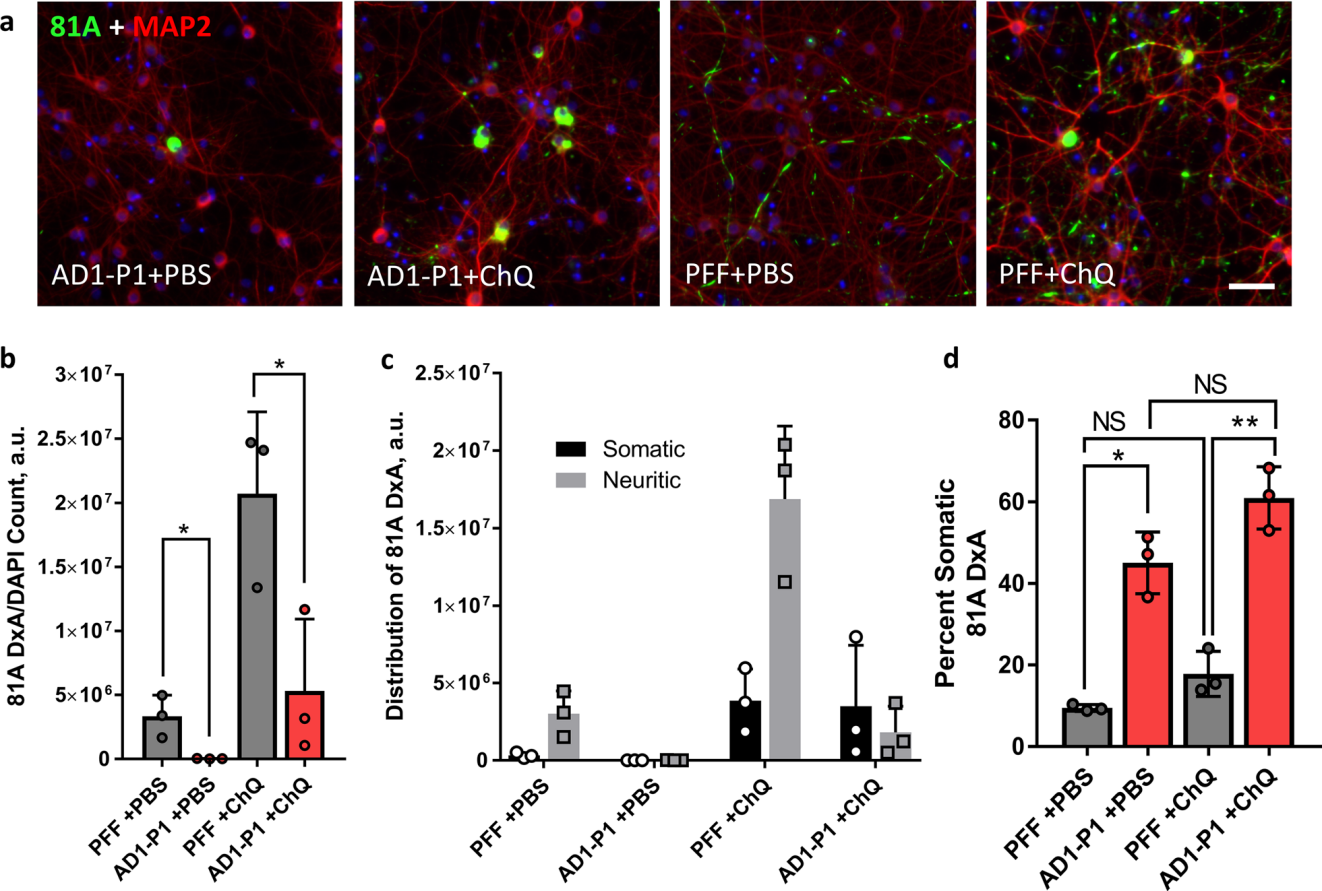
Lysosomal disruption with Chloroquine leads to increased pathology but not a change in distribution. **a** Neurons treated in 96-well plates with 25 ng per well of either AD1-P1 or PFF for 6 h followed by a 30 min pulse of either PBS or 25 uM Chloroquine (ChQ) and fixed after 10 days. Scale bar 50 µm. **b** Quantitation of total 81A signal for neurons treated as in **a**. **c** Same quantitation of total 81A as in **b**, with totals for Somatic and Neuritic pathology shown. **d** Percent of total 81A pathology in somatic inclusions from neurons treated as in **a**. Totals are the average of 3 replicate wells. Error bars represent the standard deviation. Significance compared to PFF treated neurons was determined by student t-test (*p < 0.05, **p < 0.01)

It has previously been observed that treatment with the weak, lipophilic base chloroquine (ChQ) can greatly increase the amount of α-Syn pathology induced in neurons for the same dose of PFF [27]. This is because the majority of fibrils taken up into neurons are targeted to the endosomal-lysosomal pathway and disruption of lysosomal integrity by a transient ChQ pulse presumably allows more fibrils to escape into the cytoplasm. Given that uptake of BODIPY-P1 fibrils appears to occur in both the soma and processes (Additional file 7: Fig. S7), we hypothesized that the downstream difference in pathology localization could be due to differential degradation in the lysosome. However, when neurons treated with either AD-P1 or PFF were exposed to ChQ, the result was a global increase in the amount of pathology without any change in the pattern of pathology (Fig. 6a, b). PFF treated neurons exhibited increased neuritic pathology with the addition of ChQ, while AD-P1 treated neurons exhibited more somatic inclusions but little increase in neuritic pathology following the ChQ pulse (Fig. 6c). This can also be seen when comparing the percent of somatic 81A signal between PFF and AD1-P1 with and without the ChQ pulse (Fig. 6d). This data suggests that lysosomal degradation and or escape is not responsible for the differences observed between PFF and LB-P1 pathology. If it were, disruption of the lysosome would be expected to make LB-P1 more like PFF.

## Discussion

LBs are highly complex cellular structures, and our understanding of their composition is still developing [30]. Despite their heterogeneity, we have found that LB α-Syn extracted from the brains of patients diagnosed with AD or PDD are both pathologically active in cultured mouse neurons and consistently induced a unique pathological phenotype (Fig. 2a). To our knowledge this is the first time that patient derived LB α-Syn has been shown to induce robust pathology in cultured neurons. This pathological phenotype is characterized by the predominance of large somatic inclusions and the relative lack of punctate, neuritic inclusions and is thus distinct in morphology, intensity, and distribution from α-Syn PFF induced pathology. We observed the same phenotype across cases and various doses, with the only major variation being in the intensity of the total pathology at a given dose, with some cases inducing more total pathology (Fig. 2c). While our neuron model suggests that LB and PFF α-Syn selectively induce different pathologies, we cannot rule out the possibility that the observed difference is due to selective degradation of the induced pathology. This could also explain why so many neurons in our LB treated culture have no pathology whatsoever. It has been well demonstrated recently that somatic inclusions induced by α-Syn PFF mature slowly over the lifetime of the cultured neurons and only represent a minority of the pathological inclusions [31]. Our data suggest that, by contrast, pathology induced by patient LB α-Syn is predominantly somatic from very early on (Additional file 4: Fig. S4) and thus possibly develops through a different pathway than PFF induced pathology. This is supported by our ChQ pulse experiment, wherein lysosomal disruption leads to increased pathology but no change in the distribution of the pathology, with LB-P1 + ChQ treated neurons exhibiting more somatic inclusions than LB-P1 + PBS controls, but not increasing the proportion neuritic inclusions (Fig. 6c, d).

Given the complex nature of our patient brain extracts, it is possible that there are non-α-Syn components of the extracts that are responsible for the unique LB pathology. However, this is highly unlikely for two reasons. First, the relative α-Syn concentration and total amount of protein vary widely for the extracts we tested (Fig. 1d), exposing neurons to drastically different amounts of non-α-Syn proteins for the same dose of α-Syn. Yet the pathology induced across different patient samples is largely consistent and does not scale proportionally to total protein (Fig. 2c, d). Second, when we amplified our LB α-Syn in vitro with recombinant monomer, we diluted all patient samples to the same final α-Syn concentration, resulting in a range of 5–50 fold dilution of the extract, by volume. Despite this range of dilutions, the pathology induced by the LB-P1 samples was even more consistent across all cases in both total pathology and the amount of somatic inclusions (Fig. 3e, f). Additionally, treatment with an equal dose of α-Syn PFF mixed with 5% LB seeds did not induce the same pathology as LB-P1 amplified from 5% LB seeds (Fig. 3g). These data suggest that the unique pathology we observed in LB and LB-P1 treated neurons was the result of the α-Syn aggregates themselves. Furthermore, this demonstrates that the in vitro amplification of LB α-Syn with recombinant monomer propagates the conformation essential for inducing this pathology. It has been well documented that seed-dependent amplification of α-Syn fibrils can maintain unique pathological activity [32]. PK digestion also suggests that LB and LB-P1 aggregates share a similar core conformation (Fig. 3b, c), but further biophysical characterization is necessary to demonstrate how similar they truly are. Determining whether our amplification protocol fully captures the conformation, or conformations, present in the original LB fibrils is an area of ongoing investigation. Still, we are confident that we are amplifying the conformation responsible for inducing the observed pathology.

We consider our amplification approach as complementary to recently published protein misfolding cyclic amplification (PMCA) based strategies for amplifying patient derived material [33–35]. These approaches have displayed great efficacy in distinguishing between patient and healthy brain material by amplifying minute amounts of α-Syn aggregates. They also can provide fibril preps that are relatively free from contaminant proteins, allowing for comparisons of amplified material from different synucleinopathies by spectroscopic methods. However, the small amount of primary patient derived α-Syn used in PMCA precludes direct interrogation without amplification. As a result, there is no characterization of the primary, brain-derived aggregates to use as a comparison to the resulting PMCA products. Thus while this approach can readily distinguish between patients of different diagnoses and healthy controls based on fibril production, it is not clear to what extent the fibrils that are amplified are similar to the original material. In fact, the structure of α-Syn aggregates from GCI extracted from MSA patients has been solved by cryo-electron microscopy (cryoEM) [36], but the same group found that using a PMCA approach to amplify patient GCI derived α-Syn produced aggregates with a different structure by cryo-EM [37]. This suggests that PMCA may in fact not capture the conformation of the input seeds.

An advantage to our amplification approach is that the pathology induced by the LB-P1 material can be directly compared to the pathology induced by the original seeds. This allows us to evaluate how well a given LB-P1 replicates the pathological activity of the input seeds. Future studies using a combination of these approaches could lead to additional insights on this process. The in vitro amplification approach presented here is also an ideal strategy to investigate LB α-Syn using techniques that require more material than limited patient samples can furnish without compromising biological activity. We were able to inject WT mice with a combined total of 4.5 µg of LB-P1 material while consuming only 0.225 µg total of patient LB α-Syn.

Amplifying LB α-Syn using recombinant monomer also gave us the opportunity to incorporate chemical labels or probes such as our BODIPY-LB-P1, while allowing us to confirm that the label did not alter its activity. We saw no difference between pathology of LB-P1 with or without labelled monomer, (Figs. 3d, 5b) suggesting it has not significantly perturbed the fibril conformation. Uptake of BODIPY-LB-P1 for all of the AD and PDD cases tested showed significantly more endocytosis compared to BODIPY-PFFs (Fig. 5d, e), supporting the idea that this effect is specific for LB α-Syn. This may point to additional receptors that recognize LB α-Syn but not α-Syn PFF, thus providing a greater opportunity for uptake by any given neuron. It was not apparent that this would be the case, given the less widespread and more discrete pathology observed following treatment with LB α-Syn. The distribution of BODIPY-positive fibrils does not correlate spatially with the location of the eventual Pα-Syn-positive pathology predominantly in the soma (Fig. 5f, Additional file 7: Fig S7). It has been observed that different aggregated α-Syn species can be turned over differentially by either the lysosomal or autophagic pathway [38]. It is possible then that while BODIPY-LB-P1 appears to be taken up by endocytosis throughout the neuron, it is degraded at different rates or by different pathways in the somatic and neuritic compartments. This observation could also point to alternative pathways for how cells recognize and transport LB and PFF α-Syn containing vesicles. Further investigation of labelled LB-P1 is required to dissect the mechanism that underlies the unique LB pathological phenotype.

α-Syn PFFs are an invaluable tool for modelling and understanding synucleinopathies on a cellular level. However, our data has highlighted that there are significant differences in the cellular response to patient LB material and recombinant α-Syn PFFs in cultured primary neurons. That the differences between PFF and LB extends also to in vivo experiments suggests that there may be important aspects of LB biology that α-Syn PFF based models fail to capture. We hope that further characterization of our LB-P1 aggregates will expand our ability to model the disease state of α-Syn and uncover new targets for potential therapeutic strategies.

## Supplementary Information

**Additional file 1: Supplementary Figure S1,** Ubiquitin and p62 immunohistochemistry in frontal cortex of postmortem brain samples.

**Additional file 2: Supplementary Figure S2,** LB aggregates from AD and PDD brains induce similar pathology in primary mouse neurons that are distinct from PFF pathology.

**Additional file 3: Supplementary Figure S3,** Concentration course of LB induced α-Syn pathology in primary mouse neurons.

**Additional file 4: Supplementary Figure S4,** α-Syn pathology induced by LB in primary mouse neurons is distinct from PFF even at earliest time points.

**Additional file 5: Supplementary Figure S5,** Low magnification images of LB-P1 induced α-Syn pathology in primary mouse neurons.

**Additional file 6: Supplementary Figure S6,** α-Syn pathology induced in primary mouse neurons by LB-P1 after increasing amounts of amplification.

**Additional file 7: Supplementary Figure S7,** Localization of BODIPY labelled LB-P1 in live primary mouse neurons.

**Additional file 8: Supplementary Table 1,** primary antibodies used for immunocytochemistry.

## Data Availability

All data generated or analysed during this study are included in this published article [and its supplementary information files].
